# Gambling Disorder in the United Kingdom: key research priorities and the urgent need for independent research funding

**DOI:** 10.1016/S2215-0366(21)00356-4

**Published:** 2022-04

**Authors:** Henrietta Bowden-Jones, Roxanne W Hook, Jon E. Grant, Konstantinos Ioannidis, Ornella Corazza, Naomi A Fineberg, Bryan Singer, Amanda Roberts, Richard Bethlehem, Simon Dymond, Rafael Romero-Garcia, Trevor W Robbins, Samuele Cortese, Shane A Thomas, Barbara J Sahakian, Nicki A Dowling, Samuel R Chamberlain

**Affiliations:** 1 National Problem Gambling Clinic & National Centre for Gaming Disorders, London, UK; 2 Department of Psychiatry, University of Cambridge, UK; Cambridgeshire and Peterborough NHS Foundation Trust, Cambridge, UK; 3 Department of Psychiatry and Behavioral Neuroscience, University of Chicago, Chicago, IL, USA; 4 Department of International Health, Care and Public Health Research Institute, Maastricht University, Maastricht, NL; 5 Department of Clinical, Pharmacological and Biological Sciences, University of Hertfordshire, Hatfield, UK; 6 School of Life and Medical Sciences, University of Hertfordshire, Hatfield, UK; Hertfordshire Partnership University NHS Foundation Trust, Welwyn Garden City, UK; University of Cambridge School of Clinical Medicine, Cambridge, UK; 7 School of Psychology, Sussex Addiction Research and Intervention Centre, and Sussex Neuroscience, University of Sussex, Brighton, UK; 8 School of Psychology, College of Social Science, University of Lincoln, Brayford Pool, Lincoln, UK; 9 Autism Research Centre, Department of Psychiatry, University of Cambridge, UK; Brain Mapping Unit, Department of Psychiatry, University of Cambridge, UK; 10 Department of Psychology, Swansea University, Singleton Campus, Swansea, UK; 11 Department of Psychology, Reykjavík University, Reykjavík, Iceland; 12 Department of Psychology and Behavioural and Clinical Neuroscience Institute, University of Cambridge, UK; 13 Center for Innovation in Mental Health, School of Psychology, Faculty of Environmental and Life Sciences, University of Southampton, Southampton, UK; Solent NHS Trust, Southampton, UK; Hassenfeld Children’s Hospital at NYU Langone, New York University Child Study Center, New York City, New York, USA; Division of Psychiatry and Applied Psychology, School of Medicine, University of Nottingham, UK; 14 School of Health, Federation University, Ballarat, Australia; 15 School of Psychology, Deakin University, Geelong, VIC, Australia; Melbourne Graduate School of Education, University of Melbourne, VIC, Australia; 16 Department of Psychiatry, University of Southampton, UK; Southern Health NHS Foundation Trust, Southampton, UK

**Keywords:** disordered gambling, funding, addiction, impulsive, compulsive

## Abstract

Gambling in the modern era is pervasive due to the variety of gambling opportunities including use of technology (such as online applications on smartphones). While many people gamble recreationally without undue negative impact, a sizable subset of individuals develop disordered gambling, associated with marked functional impairment including other mental health problems, relationship problems, bankruptcy, suicidality and criminality. The National UK Research Network for Behavioural Addictions (NUK-BA) was established to promote understanding, research, and treatments for behavioural addictions including Gambling Disorder, which constitutes the only currently recognized formal ‘behavioural’ addiction. This statement from NUK-BA identifies the current status of research and treatment for disordered gambling in the UK (including funding issues), and key research that must be conducted in order to establish the magnitude of the problem, vulnerability and resilience factors, neurobiology, long-term consequences, and treatment opportunities. In particular, we highlight the need to: 1) Conduct independent longitudinal research on prevalence of disordered gambling (Gambling Disorder and at-risk gambling), and gambling harms, including in vulnerable and minority groups; 2) Select and refine the optimal pragmatic measurement tools; 3) Identify predictors (vulnerability and resilience markers) of disordered gambling in people who gamble recreationally, including in vulnerable and minority groups, longitudinally; 4) Conduct randomised controlled trials (RCTs) on psychological interventions and pharmacotherapy for gambling disorder; 5) Optimise our understanding of the neurobiological basis of Gambling Disorder, including genetics, impulsivity and compulsivity, and biomarkers; and 6) Develop clinical guidelines based upon the best possible contemporary research evidence to guide effective clinical interventions. We also highlight the need to consider what can be learnt from other countries’ approaches towards mitigating gambling-related harms.

## Introduction

Gambling Disorder is a recognised mental health condition characterised by persistent and recurrent maladaptive patterns of gambling behaviour, leading to substantial functional impairment and reduced quality of life (1, 2). The primary aim of this paper is to present a consensus view from the National UK Research Network for Behavioural Addictions (NUK-BA) regarding the top unmet research priorities in the area of Gambling Disorder, and funding issues, with a UK focus. In order to achieve this aim, we first discuss contextual information about Gambling Disorder, before describing NUK-BA, outlining the state of clinical and research provision for this disorder in the UK, drawing also on international perspectives. We then present the top research priorities for Gambling Disorder in the UK and how these might be achieved.

Gambling Disorder constitutes the archetypal ‘behavioural addiction’, being the only one currently included in the same category as substance use disorders in the Diagnostic and Statistical Manual Version 5 (DSM-5) (3). Current DSM-5 criteria require endorsement of at least four of nine symptom domains for a diagnosis of Gambling Disorder. However, recent research has found that people who meet fewer diagnostic criteria (i.e., subthreshold ‘problem gambling’) nonetheless exhibit many of the negative characteristics seen with Gambling Disorder, including objective impairments in decision-making (4). Accordingly, problem gambling is often defined as gambling behaviour that leads to adverse consequences for individuals, families and communities (5), consistent with public health frameworks that conceptualise gambling problems across a continuum of risk (6). While conventionally some societal groups may not have gone into gambling arenas (e.g. betting shops or casinos), now gambling is pervasive due to online technology. It is no longer necessary to leave one’s home to gamble. Gambling Disorder appears to be more common in men compared to women, and partly distinct risk factors have been found as a function of gender (7).

People with Gambling Disorder have high rates of other (often undetected) mental health conditions including anxiety and mood disorders, substance use disorders, impulse control disorders, and attention-deficit/hyperactivity disorder (ADHD) (8–10). A systematic review and meta-analysis in treatment-seeking patients found 75% had one or more comorbidities, including nicotine dependence (56%), depression (30%) and alcohol abuse (18%) and dependence (15%) (9). There are complex bi-directional relationships between such disorders (11).

Disordered gambling can lead to financial, emotional, and relationship problems, including interpersonal violence, and – for a smaller proportion – engagement in illicit activities to fund gambling (12). As with substance use disorders, Gambling Disorder often develops during adolescence and young adulthood, and can follow a relapsing remitting course over the longer term (13). Gambling Disorder has high rates of familial transmission (1). It is also associated with considerably increased risk of suicidality, an association that is robust after controlling for relevant comorbidities (14). Additionally, some minority/vulnerable groups appear to be disproportionately affected by Gambling Disorder. For example, in a UK study, despite participation in gambling being higher in the White/White British racial-ethnic group, those from the Black/Black British racial-ethnic group were more likely to have Gambling Disorder (15); the reasons for this apparent disparity are unclear.

Studies have highlighted the importance of screening for Gambling Disorder in primary, mental health, and secondary healthcare settings, because individuals may present with other mental and physical problems (including those secondary to gambling), but often do not seek help for gambling itself, or do not mention the gambling problem without prompting (16–18). When help-seeking occurs it is typically ‘crisis driven’, only occurring after experiencing severe harm (e.g., suicide attempt) (19). Unfortunately, disordered gambling remains low on the list of priorities in UK healthcare due to lack of investment and acknowledgment, which means the true extent of gambling-related harm and the related resource pressure is ignored or unrecognised.

In the UK, the minimum legal age for most types of gambling is 18 years. In the Annual Statistics (2020) from the National Gambling Treatment Service (Great Britain), 9008 individuals were recorded as having been treated within gambling services (20). Given a conservative estimated prevalence of gambling disorder of 0.4%, and Great Britain population of approximately 50 million adults, this suggests that – as a rough estimate – less than 5% of those adults with gambling disorder received treatment by services under this framework. The treatment services currently operating in England are funded in a variety of ways: some are National Health Services (NHS) and are funded either solely or in a match funded way by NHS England funds as part of the 2019 Long Term Plan ten year strategy having included Gambling disorder as part of its remit. Other services that are non-NHS but run instead by non-statutory agencies are funded wholly by the commissioning body GambleAware, a charity set up to distribute industry donated funds designated to treatment of gambling disorder. Generally, such services offer psychotherapy for the treatment of Gambling Disorder which can range from Cognitive Behavioural Therapy, CBT in the NHS clinics to more counselling based therapies in the charitable sector.

The lack of research into gambling-related harms has been noted in the UK and contrasted to more extensive public health focus on gambling-related harms in some other countries (21). Remarkably, data on gambling-related harms across the UK and whether these are escalating longitudinally are scant. Existing data on gambling in the UK are largely from Gambling Commission reports, which focus on rates of gambling participation and problem gambling; the latter quantified using a brief rating tool or a non-validated interview tool (22). Data from 2016 indicated that 0.7% of people in England identified as problem gamblers, and 3.6% of people in England were deemed to be at low or moderate risk of developing problems with their gambling (22). However again, it is unclear how these rates map onto actual harms.

Though the current paper focuses on the situation in the UK specifically regarding clinical and research provision for disordered gambling, it should be considered that this is a global public health concern, and so the perspectives may have wider geographical implications and relevance (21). For example, a discussion paper for the World Health Organization (WHO) noted massive unprecedented increases in gambling over time, driven by online access to gambling, as well as substantial increases in disordered gambling as well as gambling-related harms, which were reported to be of similar magnitude to harms arising from depression and/or substance use problems (23). The current paper focuses on research and healthcare, but it should be noted that in the UK gambling-related harms are also a source of concern for many charitable organisations, social care, as well as politically. For example, in Britain, the House of Lords has, through the years, formed more than one evidence gathering Committee specifically looking into gambling-related issues such as gambling related societal harm (24). The current Peers for Gambling Reform is one such committee.

## National UK Research Network for Behavioural Addictions (NUK-BA) (www.nuk-ba.co.uk)

The National UK Research Network for Behavioural Addictions (NUK-BA) was established in recognition of the lack of a cohesive network to identify unmet needs in terms of research and treatment provision for behavioural addictions, including Gambling Disorder, in the UK. The network includes expertise across disciplines of public health, psychiatry, clinical psychology, neuroscience, brain imaging, genetics, study design (including longitudinal cohorts), validation of clinical rating tools, trans-diagnostic vulnerability markers, and cognitive assessment. Members are invited to join based on their expertise; and most members are based in UK, but NUK-BA also includes international members through its International Advisory Board. The group includes not only experts in addictions, but also those in complementary areas of impulsive and compulsive disorders and symptoms, such as Gaming Disorder, Problematic Usage of the Internet, ADHD, Obsessive Compulsive Disorder (OCD), eating disorders, anxiety disorders, mood disorders, and substance use disorders. In addition to expert membership in the UK, NUK-BA has an International Advisory Board to ensure work is grounded in international best practice. To maintain independence, NUK-BA is not funded by, nor does it accept funding from, industry bodies or companies.

## Current Status of Gambling Disorder Research and Treatment Services in the UK

While there is research into Gambling Disorder in the UK, it was noted following a search of traditional funding bodies that there is a marked lack of dedicated explicit independent funding for research into Gambling Disorder and related conditions in the UK. Historically, some independent bodies did provide funds for gambling research, which led to highly cited successful outcomes. Limited research funding has been available for over a decade, but linked to the gambling industry via the distributor of industry voluntary donations. Some individuals in the field argue that research funding administered through a third party dependent on the gambling industry, could be acceptable provided safeguards are in place (such as transparency, disclosures, governance procedures, and open science). However, this type of funding route is not acceptable to many in the research world (nationally and internationally) due to a number of reasons including perceived conflicts of interest and institutional rules. At present, funds for gambling research in the UK are being collected by a voluntary levy on companies, and these funds are then administered through a specific organisation whose existence is dependent on this industry funding. Many universities, researchers and clinicians in the UK cannot accept funding administered through this route. The problematic nature of this type of mechanism for administering funding has also been widely highlighted by others, including internationally (25).

In terms of UK treatment services offering evidence-based treatments for Gambling Disorder, the National Problem Gambling Clinic in London, founded in 2008 was the first NHS clinic designated specifically for the treatment of Gambling Disorder. A decade later, the NHS England Long term plan incorporated Gambling Disorder treatment into its list of services and fifteen more clinics were planned. Some are now fully functioning, including the Northern Gambling Services in Leeds. Calls have been made for the need for Gambling Disorder clinics in other parts of the United Kingdom (26). GamCare is a counselling treatment service associated with seventeen other providers across the country. Gamblers Anonymous support groups, adapted from the Alcoholics Anonymous template, are also available in the UK. The Gordon Moody Association is a charity providing residential treatment for Gambling Disorder in the UK. The specialist Impulsive and Compulsive Disorder clinic at the University of Southampton and Southern Health NHS Foundation Trust offers assessment and treatment advice to some other NHS providers, in relation to Gambling Disorder and other related conditions.

## How can research funding for Gambling Disorder best be facilitated in the UK?

It is proposed that the most efficient way to fund independent Gambling Disorder research in the UK would be to implement the 1% statutory levy placed on industry earnings that many have campaigned for in the UK Parliament (27). The money designated to gambling research each year should then be entrusted to, and administered by, a reputable independent research body unrelated to the gambling industry, such as the Medical Research Council (MRC) or another independent widely respected research charity, to distribute by placing national calls on research areas of current interest to policy, prevention, treatment and biological research. The funds should not be held or administered by any organisation that has significant potential conflicts of interest in relation to the gambling industry, such as being dependent on industry for its existence and future. Once such a suitable body to distribute the funds is identified, and confirmed as acceptable by independent experts and clinicians, it will be possible for other charitable organisations to apportion funds to research into this disorder through the same impartial mechanism.

## Top priorities for UK research into Gambling Disorder

The following top five UK research priorities were identified by the group during several UK NUK-BA meetings, through open discussion and consensus.

### 1 Conduct independent longitudinal research on prevalence of disordered gambling (Gambling Disorder and at-risk gambling), and gambling harms, including in vulnerable and minority groups

The British gambling prevalence survey of 2010 showed a prevalence of Gambling Disorder of 0.9% in the general adult population, equating to around 451,000 adults aged 16 and over in Britain (28). Less is known about gambling in adolescents. The previous surveys were conducted with similar methodologies in 2000 and 2007. These surveys were discontinued when the prevalence was seen to be increasing, at a time when the implementation of the 2005 Gambling Act in 2007 had created a deregulated market for gambling. More recent surveys show that prevalence rates remain concerningly high. For example, the Gambling Commission Report published in 2019 estimated that the rate of Gambling Disorder was 0.7%, with 3.5% displaying low- or moderate-risk gambling (29). The recent UK YouGov Gamble Aware survey indicated a prevalence of 2.7% for Gambling Disorder in adults, i.e. considerably higher than that reported in other surveys. Research indicates that even endorsement of low levels of diagnostic criteria can be associated with impairment in quality of life, similar to those seen in those meeting full diagnostic criteria for Gambling Disorder (4), highlighting the need to consider the full spectrum of symptoms at the population level.

Recent UK prevalence surveys have tended to be in relatively small samples (as compared to what would normally be regarded as acceptable for mental health prevalence studies for a country), did not include adequate independent input from diverse experts, and are likely to have under-represented vulnerable groups more exposed to developing gambling problems, such as people from certain minority ethnic groups (30). Moreover, studies have tended to be ad hoc rather than longitudinal in nature. Data from UK treatment settings indicate that the nature of gambling has changed markedly over time (e.g. growing use of online applications and forms of gambling) (31) and is now accessible by a large portion of the population anywhere and anytime. This is also likely to be the case in the general population, but is not addressed by prior prevalence surveys. As such, there is an urgent need to understand the scale of harms (including for example domestic violence, housing issues, debt, and criminal involvement) in this country attributable to gambling, in high-quality longitudinal prevalence studies that are independent, large-scale, and include input from an appropriate range of experts. This will require funding sufficient to enrol enough people to account for attrition (participants dropping out of such surveys) in longitudinal research over time.

Another possibility to help partly achieve this goal could be to leverage existing large-scale datasets in the UK, i.e. register-based data studies, when they include information about gambling disorder (e.g. see 32). The use of register data has proven very fruitful in longitudinally examining other areas of mental health in the UK e.g. (33), as well as in charactering gambling problems in other countries such as in Norway (34). Of course, this would be contingent on collecting appropriate measurements, which is often not the case for such areas of mental health symptoms.

### 2 Select and refine the optimal pragmatic measurement tools

A variety of self-rated and clinician-administered instruments are available to assess Gambling Disorder and at-risk gambling, but there is a lack of consensus on the most suitable tool(s) to be used for specific contexts (35). In particular, it is likely that different optimal pragmatic measurement tools will be needed for: (i) diagnosing disordered gambling and measuring symptom severity; (ii) screening for disordered gambling; (iii) measuring treatment response; (iv) measuring gambling-related harm; and (v) screening for the most relevant comorbidities.

For example, in terms of procedures used in prevalence surveys in the UK, some refer to using the ‘DSM diagnostic criteria’ itself to identify Gambling Disorder, but this approach has unknown reliability and validity and depends on the exact form used. The DSM criteria themselves are not a structured clinical interview, they are simply a list. The gold standard would be a structured clinical interview that has been previously rigorously validated, but of course this is relatively labour intensive and requires adequate funding to be available, including to train raters and monitor quality. Validated clinical interviews exist such as the Minnesota Impulse Disorder Inventory (MIDI) (36), but have generally not been included in these prevalence surveys. Some UK surveys used the Problem Gambling Severity Index (PGSI), which has good properties for identifying Gambling Disorder, but is less suitable for differentiating between milder potentially clinically relevant forms of gambling problems (37). Also, such instruments do not provide information on the precise forms of gambling (e.g. casinos, racing, online, bingo, etc.), which have already been reported to change markedly over time (31). They have been inappropriately employed in clinical settings to measure response to treatment (38), when they are not validated for that purpose.

Another consideration is that many instruments have not been thoroughly psychometrically validated in UK populations. Psychometric scale properties, including optimal thresholds to determine ‘caseness’ or severity, can differ across countries, e.g. (39). This is likely to be particularly the case for gambling where its forms are likely to be very culture dependent. Additionally, many instruments have not been subjected to more rigorous statistical validation procedures such as statistical Item Response Theory (IRT) analysis (40).

The application of the public health framework to gambling has also resulted in growing concern that gambling research, and subsequent gambling policy, has conflated problem gambling severity and gambling-related harm (41). In recognition that problem gambling severity and harm are closely coupled, but conceptually distinct, constructs, instruments specifically measuring gambling-related harm are emerging (42), but these have not, to date, been employed in the UK prevalence surveys.

Brief screening instruments, which are necessary to facilitate the early identification of disordered gambling in clinical and research settings, are increasingly available but few can satisfactorily identify both at-risk and problem gambling (35). Moreover, Gambling Disorder is highly comorbid with impulsive and compulsive conditions, such as ADHD, and formal impulse control and obsessive-compulsive disorders, but screening for these does not often occur (36, 43). This means that the contribution and role of other common disorders cannot be evaluated.

Therefore, we need for a range of experts to identify and refine the most optimal tools for UK research, for specific purposes, based on detailed psychometric analyses with the latest statistical approaches.

### 3 Identify predictors (vulnerability and resilience markers) of disordered gambling in people who gamble recreationally, including in vulnerable and minority groups, longitudinally

Some research has been conducted on longitudinal trajectories of gambling in young people, including candidate vulnerability and resilience markers (44). Because many young people who gamble later stop gambling, show remission of sub-syndromal disordered gambling, and/or are lost to follow-up, large sample sizes are needed in order to identify such markers. For example, in a US study following 575 non-treatment seeking young adults over three years, three latent subtypes were identified: a high harm group (N = 5.6%) who had moderate-severe Gambling Disorder at baseline, remaining symptomatic at follow-up; an intermediate harm group (19.5%) who had problem gambling reducing over time; and low harm group (75.0%) who were essentially asymptomatic over time (45). The non-low-harm groups had higher traits of impulsivity and compulsivity, cognitive deficits, and higher rates of mental disorders at baseline (including substance use). These results suggest the existence of distinct resilience/vulnerability trajectories in the natural history of GD and thus highlight the need for a richer understanding of antecedents at multiple bio-psycho-social levels (46).

Therefore, what is needed is large scale longitudinal UK research that includes measurement domains already implicated in the manifestation of disordered gambling, such as childhood experiences (trauma, parenting, friendship groups), antisocial behaviours, impulsive and compulsive traits, cognition, and comorbid mental health and substance use problems (44). Moreover, given that the majority of the available research examines individual vulnerability markers, there is a need for future longitudinal research to investigate resilience markers, as well as vulnerability markers across relationship, community, and societal domains (44). Such research should consider pathways to gambling, including – for example – the role of Internet Gaming Disorder or Problematic Usage of the Internet (47) – via targeted advertising of vulnerable groups, but also due to the given trends in the respective industries for the “gamblification” of online gaming and “gamification” of online gambling (48).

Bearing in mind that approximately 5.6% of people who gamble recreationally are likely to fit the ‘high harm’ group based on the above US research, power calculations can be conducted to ensure such a longitudinal study would be sufficiently large to address this issue. Because of the large sample sizes needed, such research is likely to require the use of Internet-enabled data including self-report questionnaires and validated online cognitive tests (36, 49–53). These approaches have been shown to be valuable when used at scale to monitor mental health and other consequences of the COVID-19 pandemic (54).

### 4 Conduct Randomised Controlled Trials (RCTs) on psychological interventions and pharmacotherapy for gambling disorder

Data collected over the past 20 years support the use of some sort of cognitive behavioural therapy (CBT) and motivational interviewing (MI) for problem gambling (55). CBT has taken many forms including individual cognitive therapy, group or individual CBT, use of imaginal desensitization, and brief interventions using bibliotherapy (56–58). In addition, several double-blind, placebo-controlled studies support the use of the opioid antagonist, naltrexone, in reducing gambling urges and behaviour (1, 59, 60). Taken together, however, there has been little research on what predicts who will benefit from these options, what variables hinder successful outcomes (61) and the neurobehavioural mechanisms by which treatments achieve remission and whether other forms of pharmacotherapy might also be effective. There are also virtually no data regarding how much therapy is ideally needed by any single individual, or how treatment options should be sequenced. Thus, a need for personalized medicine in the area of gambling treatment is urgently needed.

Additionally, the role of innovative treatment approaches should be considered, including the application of transdiagnostic approaches [potentially targeting common features across disorders (62)], third-wave CBT interventions (such as mindfulness and acceptance-based approaches) (63) and neurocognitive interventions (64), non-invasive neuromodulation, and the potential for digital tools to support stimulus control in treatment and to deliver early phase interventions (65, 66). There is a poverty of studies in this area but lessons can be learned from the use of such approaches for other conditions, notably substance use disorders (67, 68).

### 5 Optimise our understanding of the neurobiological basis of Gambling Disorder, including genetics, impulsivity and compulsivity, and biomarkers

Much of our understanding of neurobiology involved in Gambling Disorder is informed by the larger body of research that is available from the studies of substance use disorder, including alcohol. However, relative to other areas of mental health, understanding of Gambling Disorder is relatively limited. By way of example, a PubMed search for papers using the keywords “gambling” and “neuroimaging” yields just 761 results to date, as compared to 4898 results for “alcohol” and “neuroimaging”.

In broad terms, Gambling Disorder has been associated with abnormalities of brain reward pathways (including the striatum) (69), as well as with cognitive problems reflecting loss of top-down control over urges and habits, subserved by parts of the cortex, especially prefrontal cortex (52, 70, 71). Interestingly, despite clinical parallels (72), differences are emerging in the patterns of brain abnormalities reported between Gambling Disorder and alcohol use disorder (73), though, of course, direct comparison is problematic since substance intake has complex and different acute, sub-chronic, and chronic effects on the brain. As highlighted earlier, brain research into Gambling Disorder often does not measure and control for the influence of other disorders, such as attention-deficit hyperactivity disorder (ADHD), impulse control problems, compulsive symptoms, and substance use.

The neurobiology of Gambling Disorder should also be informed by other areas of investigation, drawing together experts from traditionally disparate disciplines. A key example here would be of the study of Gambling Disorder, and impulse control disorders, in patients with Parkinson’s Disease. There is evidence of comorbid overlap between these conditions, including in a subset of patients treated with dopaminergic medications (74–76). In turn, this has potential implications for understanding the role of dopamine in Gambling Disorder per se.

International efforts have also been building towards trans-diagnostic tools to identify factors that contribute to a variety of addictive problems (77, 78). By identifying common risk factors, we may be able to intervene to reduce harms not only related Gambling Disorder, but also other impulsive and compulsive problems. The concepts of impulsivity and compulsivity are likely to be important in the search for relevant biomarkers for Gambling Disorder, both in terms of vulnerability and chronicity (78). Validated self-report tools to quantify impulsivity have long-existed, and now there are also validated tools to measure compulsivity trans-diagnostically (40). Ultimately, we need studies that incorporate an appropriate comprehensive range of measures in one setting, including genetics, blood markers, cognition, and (ideally) neuroimaging.

### 6 Develop clinical guidelines based upon the best possible contemporary research evidence to guide effective clinical interventions

We note the Australian NHMRC clinical guideline for problem gambling (79) and the NICE progress towards the development of a UK guideline for disordered gambling. The integration of high-quality evidence into such guidelines and their translation into clinical practice for behavioural addictions ensures that treatment participants are given the best possible chance of recovery from their conditions. It will be important to ensure appropriate representation in terms of gender, ethnicity, culture, and age, when developing guidelines; as well as sufficient breadth of gambling research and clinical expertise.

## Concluding remarks

Gambling Disorder is responsible for significant personal, societal, financial, and professional harms in the UK and globally, but our understanding of the prevalence and course of this disease is limited, due to the severe dearth of high quality independently funded research into this area; as well as by a lack of appropriate expert consultation. For example, the Gambling Commission in their recent consultation stated that research and other experts had been consulted, but in our survey across the whole NUK-BA group, no members reported having been consulted as part of this process at all. Unless consultations encompass national and international experts in the field, policy and research limitations will continue to be propagated.

This paper has focused on Gambling Disorder. However, there is a far greater proportion of the population being impacted negatively by gambling harms than just the people with Gambling Disorder. The true extent of the harms incurred by families, spouses, employers, must also not be forgotten when research is being commissioned. Gambling in a sub-syndromal way (i.e. endorsement of some diagnostic criteria, but falling short of current diagnostic threshold) is also associated with significant harm, and will impact on a much greater proportion of the population. This too requires scrutiny. What is needed is a comprehensive examination of the full spectrum of severities and types of gambling. Furthermore, it is important to consider what can be learnt from experiences in other countries, some of which have used very different approaches to the UK in regulating gambling (e.g., Finland), and/or given greater consideration to measuring and minimising gambling-related harms (e.g., Australia).

The National UK Research Network for Behavioural Addictions (NUK-BA) provides a collaborative, inclusive framework and impetus for independent research into gambling and other behavioural addictions in the UK. This group is comprised of experts from diverse disciplines, bringing together the country’s most established researchers and clinicians in the field of behavioural addictions (80). International experts are highly valued members of the group and bring objectivity to our position statements and work. In parallel, it is important to bear in mind potential limitations of the current position paper. First, it reflects the collective perspectives of NUK-BA membership and so views of other individuals and organisations may differ. Perspectives of other experts are also important. Second, the top priorities are a consensus perspective rather than being generated through a formal research methodology e.g. Delphi. Such a position paper may in fact set the ground and inform a future Delphi study. Third, the paper focuses mainly on the clinical/research aspects rather than wider aspects of social care, charitable work, or political debate, around minimising gambling-related harms; and also provides a UK-specific focus, rather than a detailed overview of the situation in other counties.

Without adequate independently administered funding designated for research into behavioural addictions in the UK, it is likely that the UK will continue to fall short of what is achievable. The time has come for scholars to receive the necessary independent resources, allowing them to produce high quality studies this country urgently needs. The UK is fortunate to have world famous researchers in addiction, including behavioural addictions, to develop more effective treatments and to train young scientists in the field. Given sufficient Government and independent funding specifically designated for behavioural addition research, there is much that can be achieved. A targeted and strategic effort is now required to prevent problem gambling in young people and improve the quality of life and wellbeing of those recovering from behavioural addictions and their families.
